# Genetic variants in telomere‐maintenance genes are associated with ovarian cancer risk and outcome

**DOI:** 10.1111/jcmm.12995

**Published:** 2016-11-07

**Authors:** Yuhui Sun, Wade Tao, Maosheng Huang, Xifeng Wu, Jian Gu

**Affiliations:** ^1^ Department of Obstetrics and GynecologyThe First Affiliated Hospital of Harbin Medical UniversityHarbinChina; ^2^ Department of EpidemiologyThe University of Texas MD Anderson Cancer CenterHoustonTXUSA

**Keywords:** single nucleotide polymorphism, ovarian cancer, telomere maintenance, cancer risk, survival, therapeutic response

## Abstract

Most ovarian cancer patients present at an advanced stage with poor prognosis. Telomeres play a critical role in protecting chromosomes stability. The associations of genetic variants in telomere maintenance genes and ovarian cancer risk and outcome are unclear. We genotyped 137 single nucleotide polymorphisms (SNPs) in telomere‐maintenance genes in 417 ovarian cancer cases and 417 matched healthy controls to evaluate their associations with cancer risk, survival and therapeutic response. False discovery rate *Q*‐value was calculated to account for multiple testing. Eleven SNPs from two genes showed nominally significant associations with the risks of ovarian cancer. The most significant SNP was *TEP1*: rs2228026 with participants carrying at least one variant allele exhibiting a 3.28‐fold (95% CI: 1.72‐6.29; *P* < 0.001, *Q* = 0.028) increased ovarian cancer risk, which remained significant after multiple testing adjusting. There was also suggested evidence for the associations of SNPs with outcome, although none of the associations had a *Q* < 0.05. Seven SNPs from two genes showed associations with ovarian cancer survival (*P* < 0.05). The strongest association was found in *TNKS* gene (rs10093972, hazard ratio = 1.88; 95% CI: 1.20‐2.92; *P* = 0.006, *Q* = 0.076). Five SNPs from four genes showed suggestive associations with therapeutic response (*P* < 0.05). In a survival tree analysis, *TEP1*:rs10143407 was the primary factor contributing to overall survival. Unfavourable genotype analysis showed a cumulative effect of significant SNPs on ovarian cancer risk, survival and therapeutic response. Genetic variations in telomere‐maintenance genes may be associated with ovarian cancer risk and outcome.

## Introduction

Ovarian cancer is the most frequent cause of cancer‐related death among gynaecological malignancies. In 2015, the estimated new cases were 21,290 in the United States, and the estimated deaths were 14,180 1. Non‐Hispanic Whites have the highest incidence rate of ovarian cancer in the U.S. Current surveillance strategy, by trans‐vaginal ultrasound and serum tumour marker cancer antigen 125 (CA_125_), is ineffective in detecting ovarian cancer at an early stage 2, 3. As a result of the absence of obvious clinical symptoms and sensitive screening tests, most ovarian cancer patients (61%) are diagnosed at advanced stages. The 5‐year relative survival rates of ovarian cancer patients with local, regional and distant stages are 92.3%, 71.7% and 27.4% respectively 4.

The aetiology of ovarian cancer remains poorly understood. Many factors are thought to be associated with ovarian cancer, including smoking, infertility, endometriosis, oestrogen use for menopause hormone therapy, family ovarian cancer history, Lynch syndrome and mutations in *BRCA1* or *BRCA2* genes 5, 6, 7. Genome‐wide association studies (GWAS) have identified a number of susceptibility loci for ovarian risk and clinical outcome 8, 9, 10, 11, 12, 13. Previous candidate gene studies also reported nucleotide excision repair pathway, microRNA biosynthesis pathway, transforming growth factor*‐*β pathway and fibroblast growth factor pathway genetic variants may be associated with ovarian cancer risk or clinical outcome 14, 15, 16, 17.

Telomeres are nucleoprotein complexes at the ends of chromosomes and consist of short repetitive sequences (TTAGGG in humans) and a set of specialized proteins 18. Telomeres play a critical role in protecting chromosomes from degradation, end‐to‐end fusion, abnormal recombination and other detrimental chromosomal events. In normal somatic cells, telomeres are progressively eroded by 30–200 bp during each mitotic cell division 19. Many proteins are involved in regulating telomere functions. When telomere lengths become critically short, the process of cell senescence is initiated, resulting in cell‐cycle arrest or apoptosis in normal cells 20. Telomerase is activated in the majority of cancer cells that compensates telomere erosion and gives cancer cells growth advantage 21. Telomere shortening and telomerase activation are hallmarks of human cancers. Higher telomerase activity has been observed in poorly differentiated ovarian tumours tissue 22, 23. Several studies have suggested that leucocyte telomere length is associated with ovarian cancer risks 24, 25, 26. Genome‐wide association studies and candidate gene study have shown that single nucleotide polymorphisms (SNPs) in human telomerase reverse transcriptase (hTERT) gene were associated with the risk of ovarian cancer 27, 28, 29, 30. There were scarce studies evaluating the associations of hTERT and other telomere‐maintenance genes with ovarian cancer outcome. One study of 40 tagging SNPs from five telomere‐maintenance genes showed no associations between these SNPs and ovarian cancer survival, but there were some suggestive associations in subgroup analyses 31.

We hypothesize that common SNPs in telomere‐maintenance genes are significantly associated with ovarian cancer risk, survival and therapeutic response. We used a case–control study to test our hypothesis.

## Materials and methods

### Study population

Patients (*n* = 417) with pathologically confirmed ovarian cancer were recruited from the University of Texas MD Anderson Cancer Center from 1998 to 2011. All case participants were newly diagnosed, histologically confirmed ovarian cancer and previously untreated before enrolment. There were no age, ethnicity or cancer stage restrictions on recruitment. Healthy control participants (*n* = 417) were recruited from Kelsey‐Seybold Clinic, a large multi‐specialty physician group in Houston metropolitan area. Controls without cancer history other than non‐melanoma skin cancer were recruited during the same time period as the cases, and were matched to cases on age (±5 year) and ethnicity. All study participants signed written informed consent before participation. The study was approved by the institutional review boards of MD Anderson and Kelsey‐Seybold Clinic. Informed consents were obtained from all participants. Epidemiologic data including demographics, tobacco use history, bw and height, history of cancer, and medical history were collected for all cases and controls. Information on vital status was obtained from the medical records and the Social Security Death Index. For each participant, a blood sample was drawn into coded heparinized tubes for lymphocyte isolation and DNA extraction.

### SNP selection and genotyping

The details of SNP selection and array construction were described in our previous publication 32. Briefly, selected tagging SNPs have an *r* ^2^ threshold of 0.8 and minor allele frequency (MAF) greater than 0.05 in Caucasians. For each gene with a high priority score, we identified the tagging SNPs ranging from 10 kb upstream of transcriptional start site to 10 kb downstream of translational end site 33. We also identified potentially functional SNPs, which are located in the functional region of genes, including coding SNPs (synonymous SNPs and non‐synonymous SNPs) and regulatory [promoter, splicing site, 5′ untranslated region (5′UTR) and 3′UTR] regions. A complete set of SNPs was sent to Illumina technical support for custom iSelect, Infinium II BeadChip design using a proprietary program developed by Illumina. A total of 145 SNPs from 11 telomere‐maintenance genes were identified. The number of SNPs for each gene regions was as follows: *PINX1*, 27; *PTOP*, 4; *POT1*, 7; *TEP1*, 46; *TERF1*, 5; *TERF2*, 4; *TERF2IP*, 4; *TERT*, 15; *TNKS*, 21; *TNKS1BP1*, 6; and *TNKS2*, 6. Genomic DNA was extracted from peripheral blood lymphocytes using QIAmp DNA extraction kit (Qiagen, Hilden, Germany) and genotyped according to the standard protocol for Illumina's Infinium iSelect HD custom Genotyping Beadchip provided by Illumina (San Diego, CA, USA). The genotypes were auto‐called using the BeadStudio software. All the laboratory personnel performing the experiments described above were blinded to the case–control and outcome status of the DNA samples. All the laboratory personnel performing genotyping were blinded to the case–control and outcome statuses.

### Statistical analysis

Statistical analysis was performed using STATA software (version 10; STATA Corporation, College Station, TX, USA). The difference between participant groups with regard to categorical variables was compared by either Pearson's chi‐squared test or Fisher's exact test. Student's *t*‐test was used to assess continuous variables. Among the control participants, goodness‐of‐fit chi‐squared analysis was used to test Hardy–Weinberg equilibrium to each SNPs. Unconditional logistic regression was used to estimate the odds ratio (OR) and 95% confidence interval (CI), adjusting for age, smoking status and body mass index (BMI). Three genetic models (dominant, recessive and additive) were tested for each SNPs and the model with the highest significance was used to determine the statistical significance of each SNP 34. Overall survival (OS) was calculated from the date of diagnosis to the date of death or the end of patient follow‐up. The effects of SNPs on ovarian cancer survival were estimated as hazard ratios (HR) and 95% confidence intervals (95% CI) using multivariate Cox proportional hazards regression analysis. The co‐variants included were age, histology, clinical stage, tumour grade and treatment information. Kaplan–Meier curves and log‐rank tests were used to compare the OS differences by different genotypes. Higher order gene–gene interactions were explored using the Classification and Regression Tree analysis, performed using HelixTree Software (Golden Helix, Bozeman, MT, USA). Survival tree analysis was performed using the STREE program (http://masal.med.yale.edu/stree/), which also uses recursive partitioning method. Platinum‐based therapeutic response was defined by whether there was evidence of residual disease as determined by various clinical measures, such as positron emission tomography and computed tomography scans, second‐look surgery and post‐chemotherapy CA_125_ level. For response to therapy, unconditional multivariate logistic regression analysis was used while adjusting for age, histology, clinical stage, tumour grade and therapeutic information. Cumulative effects of multiple unfavourable genotypes were evaluated by counting the number of unfavourable genotypes from SNPs identified from in the main analysis (*P* < 0.05). According to the tertile distribution, the unfavourable genotypes were collapsed into high, medium and low groups. All *P*‐values reported were two‐sided. *P* < 0.05 was considered statistically significant. As an adjustment for multiple testing, false discovery rate (FDR) based *Q*‐value was calculated for each SNP using the R‐package 35. As previously suggested, we reported all those SNPs with *Q* < 0.20 to account for multiple testing while balancing the discovery nature of the study 36.

## Results

### Characteristics of the study population

Details regarding participant recruitment and participant characteristics have been described in previous publications (Table S1) 14. Briefly, a total of 417 case participants and 417 control participants were included. The cases and controls were matched on age (mean ± S.D., 60.7 ± 10.4 *versus* 60.3 ± 10.7; *P* = 0.554). Because of the small number of participants from other ethnicities, statistical analyses for overall risk assessment were restricted to 338 Caucasian cases (81.3%) and 349 Caucasian controls (83.7%). For clinical outcome analyses, we only focused on patients who had received surgery and platinum‐based chemotherapy to minimize treatment effects on survival. Among this group, 87.8% were in at advanced stages (III–IV), 46% (*n* = 146) of the patients had died at the end of the follow‐up period with 48% (*n* = 152) showing cancer recurrence and 33% (*n* = 105) being non‐responders to treatment. The median survival time (MST) was 48.3 months.

### SNPs in the telomere‐maintenance genes associated with overall ovarian cancer risk, survival and therapeutic response

Among the genotyped 145 SNPs, 11 SNPs from two genes (TEP1 and TERT) showed significant associations with overall risk of ovarian cancer (*P* < 0.05 and *Q* < 0.10; Table 1). The most significant SNP was *TEP1*: rs2228026 with participants carrying at least one variant C allele exhibiting a 3.28‐fold (95% CI: 1.72–6.29; *P* < 0.001, *Q* = 0.028) increased ovarian cancer risk.

**Table 1:** Genes and SNPs in telomere‐maintenance genes associated with overall risk of ovarian cancer

Gene	Chr	SNP	Genotype	Position	dbSNP	MAF		Model*	OR† (95% CI)	*P*‐value	*Q*‐value
					Func annot	Case	Control				
TEP1	14	rs2228026	T>C	20864049	Ile573/synonymous	0.056	0.019	DOM	3.28 (1.72–6.29)	<0.001	0.028
TEP1	14	rs2228042	G>A	20844383	The2043/synonymous	0.075	0.036	DOM	2.19 (1.32–3.64)	0.002	0.075
TEP1	14	rs4246977	T>C	20882591	5′UTR	0.353	0.413	ADD	0.55 (0.36–0.84)	0.006	0.075
TEP1	14	rs2228041	G>A	20852267	Arg1155Gln/missense	0.063	0.032	DOM	2.11 (1.23–3.61)	0.007	0.075
TEP1	14	rs2229101	T>G	20845521	Leu2002/synonymous	0.072	0.037	DOM	2.00 (1.21–3.31)	0.007	0.075
TEP1	14	rs938887	A>G	20847202	Asp1730/synonymous	0.215	0.185	ADD	3.88 (1.42–10.59)	0.008	0.075
TEP1	14	rs1713418	T>C	20834809	3′UTR	0.490	0.418	REC	1.33 (1.08–1.65)	0.009	0.075
TEP1	14	rs2297612	A>T	20869819	Intron	0.470	0.426	DOM	1.55 (1.12–2.16)	0.009	0.075
TEP1	14	rs1713456	C>T	20850093	Cys1468Tyr/missense	0.200	0.145	DOM	1.53 (1.11–2.11)	0.010	0.076
TEP1	14	rs1713436	C>T	20883949	5′UTR	0.133	0.096	DOM	1.61 (1.11–2.32)	0.012	0.083
TERT	5	rs2853676	G>A	1288547	Intron	0.308	0.259	ADD	2.32 (1.25–4.28)	0.007	0.075

*Models of inheritance: Add‐additive; Dom‐dominant; Rec‐recessive. ^†^Adjusted for age, smoking and body mass index.

Seven SNPs from two genes (TEP1 and TNKS) showed significant associations with ovarian cancer survival (*P* < 0.05, *Q* < 0.10; Table 2). The variant C allele of *TEP1*: rs938887 was associated with a 2.39‐fold increased risk of death during follow‐up period (95% CI: 1.22–4.66; *P* = 0.011). The variant C allele of *TEP1*: rs1713423 were associated with a decreased risk of death (HR: 0.53; 95% CI: 0.34–0.85; *P* = 0.008).

**Table 2:** Genes and SNPs in telomere‐maintenance genes associated with overall survival of ovarian cancer

Gene	Chr	SNP	Genotype	Position	dbSNP	MAF		Model*	HR† (95% CI)	*P*‐value	*Q*‐value
					Func annot	Case	Control				
TEP1	14	rs10143407	C>G	20831794	3′UTR	0.494	0.516	DOM	2.01 (1.21–3.32)	0.007	0.076
TEP1	14	rs1713423	T>C	20860073	Intron	0.083	0.089	ADD	0.53 (0.34–0.85)	0.008	0.076
TEP1	14	rs2151753	G>A	20880328	Intron	0.158	0.135	DOM	1.57 (1.09–2.26)	0.015	0.076
TEP1	14	rs938887	A>G	20847202	Asp5230/synonymous	0.215	0.185	ADD	2.39 (1.22–4.66)	0.011	0.076
TNKS	8	rs10093972	T>C	9451369	Intronic	0.046	0.068	DOM	1.88 (1.20–2.92)	0.006	0.076
TNKS	8	rs33944167	G>A	9564485	Pro1439/synonymous	0.069	0.072	DOM	1.81 (1.13–2.91)	0.014	0.076
TNKS	8	rs6990116	T>C	9577096	Intron	0.074	0.083	DOM	1.83 (1.15–2.92)	0.011	0.076

*Models of inheritance: Add‐additive; Dom‐dominant; Rec‐recessive. ^†^Adjusted for age, histology, clinical stage, tumour grade and treatment.

For response to platinum‐based adjuvant chemotherapy, four SNPs from three genes showed significant association (*P* < 0.05, *Q* < 0.20; Table 3). *PINX1:* rs7826180 displayed the greatest risk for poor treatment response. The variant A allele of *PINX1:* rs7826180 was associated with a 6.77‐fold increased risk of poor response to chemotherapy (95% CI: 1.68–27.27; *P* = 0.007).

**Table 3:** Genes and SNPs in telomere‐maintenance genes associated with response to treatment of ovarian cancer

Gene	Chr	SNP	Genotype	Position	dbSNP	MAF		Model*	OR† (95% CI)	*P*‐value	*Q*‐value
					Func annot	Case	Control				
TEP1	14	rs10143407	C>G	20831794	3′UTR	0.046	0.068	DOM	3.79 (1.53–9.37)	0.004	0.158
TEP1	14	rs2151753	G>A	20880328	Intron	0.158	0.135	DOM	2.17 (1.24–3.81)	0.007	0.158
PINX1	8	rs7826180	G>A	10627844	Intron	0.237	0.255	ADD	6.77 (1.68–27.27)	0.007	0.158
TERF2IP	16	rs7193066	A>C	75689521	Intron	0.050	0.057	DOM	2.98 (1.30–6.82)	0.010	0.158

*Models of inheritance: Add‐additive; Dom‐dominant; Rec‐recessive. ^†^Adjusted for age, histology, clinical stage, tumour grade and treatment.

### Cumulative effects of unfavourable genotypes in the telomere‐maintenance genes on ovarian cancer risk, treatment response and survival

We then performed cumulative unfavourable genotype analyses. In the cancer risk analysis, because *TEP1*: rs2228042 and rs2229101 exhibited high linkage, the former was included in the analysis. Compared to individuals with 0–2 unfavourable genotypes, those with 3 unfavourable genotypes and 4–8 unfavourable genotypes had a 1.63‐fold (95% CI: 1.11–2.41; *P* = 0.013) and 2.94‐fold (95% CI: 2.03–4.26; *P* = 1.26 × 10^−8^) increased risks, respectively, c (*P*_trend_ = 1.05 × 10^−8^; Table 4).

**Table 4:** SNPs associated with overall ovarian cancer risk, risk of death and risk of poor response by unfavourable genotype analysis

Risk group	Low	Medium	High	*P*_trend_
Overall risk of ovarian cancer				
No. of unfavourable genotypes	0–2	3	4–8	
Case (%)	88 (27.8)	87 (27.4)	142 (44.8)	
Control (%)	162 (46.4)	98 (28.1)	89 (25.5)	
OR* (95% CI)	1 (reference)	1.63 (1.11–2.41)	2.94 (2.03–4.26)	
*P*‐value		0.013	1.26 × 10^−8^	1.05 × 10^−8^
Overall risk of death				
No. of unfavourable genotypes	0	1	2‐4	
Dead (%)	14 (9.6)	62 (42.5)	70 (47.9)	
Alive (%)	28 (16.2)	97 (56.1)	48 (27.7)	
HR† (95% CI)	1 (reference)	1.07 (0.59–1.96)	2.88 (1.59–5.23)	
*P*‐value		0.821	4.98 × 10^−4^	6.58 × 10^−7^
Overall risk of poor response				
No. of unfavourable genotypes	0	1	2	
Non‐response (%)	36 (37.5)	42 (43.8)	18 (18.8)	
Response (%)	135 (67.8)	54 (27.1)	10 (5.0)	
OR† (95% CI)	1 (reference)	3.06 (1.70–5.51)	8.33 (3.26–21.29)	
*P*‐value		1.98 × 10^−4^	9.60 × 10^−6^	3.99 × 10^−7^

*Adjusted for age, smoking status and BMI. ^†^Adjusted for age, histology, clinical stage, tumour grade and treatment.

For treatment response, patients carrying one or two unfavourable genotypes had significantly worse response (OR = 3.06; 95% CI: 1.70–5.51, *P* = 1.98 × 10^−4^ and OR = 8.33; 95% CI: 3.26–21.29; *P* = 9.60 × 10^−6^, respectively) compared to the reference group of patients without any unfavourable genotype (*P*_trend_ = 3.99 × 10^−7^; Table 4).

For OS, because *TNKS*: rs10093972, rs33944167 and rs6990116 exhibited high linkage, rs10093972 was included in analysis together with the 4 TEP1 SNPs (Table 2). Compared to patients without unfavourable genotype, patients carrying 1 and 2–4 unfavourable genotypes had increased risks of death with HRs of 1.07 (95% CI: 0.59–1.96; *P* = 0.821) and 2.88 (95% CI: 1.59–5.23; *P* = 4.98 × 10^−4^) respectively (Table 4).

**Figure 1:**
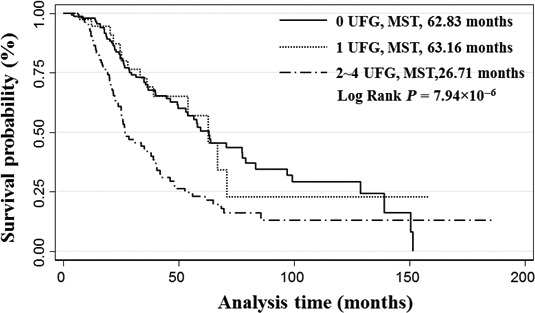
Kaplan–Meier curve of ovarian cancer patient with different unfavourable genotype. Patients were classified into three different groups based on the number of unfavourable genotypes (UFG) in each patient that was identified from cumulative effect analysis. The median survival time (MST) of each group was compared by the log‐rank test.

We also performed Kaplan–Meier curves and log‐rank tests to compare OS differences in patients with different unfavourable genotypes. The results showed a trend towards decreased survival with increasing number of unfavourable genotypes. The MST for patients with 2–4 unfavourable genotypes was 26.7 months compared to 62.8 months for those without unfavourable genotype (*P* = 7.94 × 10^−6^, log‐rank test; Fig. 1).

**Figure 2:**
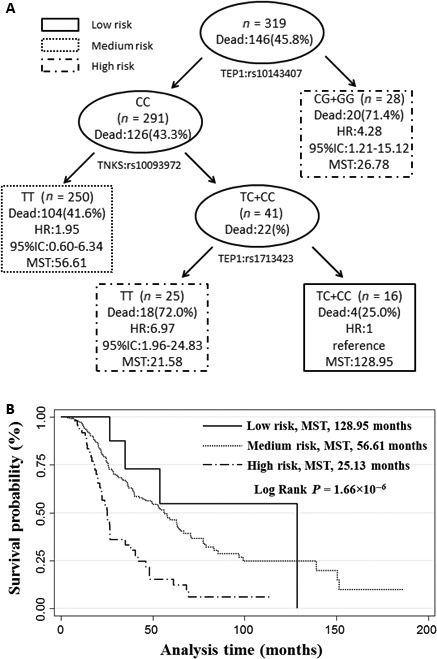
High‐order interactions of SNPs on modulating the overall survival of ovarian cancer patients. (**A**) Survival tree using a recursive partitioning method implemented in the STREE program to split the patients into nodes with different risks of death based on the distinct genotype combinations; (**B**) Kaplan–Meier curve of ovarian cancer patients with distinct genotype combinations. The terminal nodes identified in **A** were classified into three risk groups (low, medium and high) based on the HR of the terminal nodes. The median survival time (MST) of each group was compared by the log‐rank test.

We also performed a survival tree analysis for these seven variants (Fig. 2). The first split on the survival tree was *TEP1:*rs10143407, indicating that this SNP is the primary factor contributing to OS. When we used individuals of terminal node 2 as reference, the HRs for the other three terminal nodes ranged from 1.95 to 6.97 (Fig. 2A). Classifying these terminal nodes into three groups (low, medium and high), the MST for patients in the low‐risk, medium‐risk and high‐risk groups were 128.9, 56.6 and 25.1 months respectively (*P* = 1.66 × 10^−6^, log‐rank test; Fig. 2B).

## Discussion

In this study, we took a pathway‐based approach to comprehensively investigate the associations of genetic variants in telomere‐maintenance genes with ovarian cancer risk and outcome. Eleven SNPs from two genes showed significant associations with overall ovarian cancer risk, 10 of which were located on *TEP1* gene. The most significant SNP was *TEP1*: rs2228026, which remained significant after adjusting for multiple testing. *TEP1*: rs2228026 is a synonymous SNP located in the exon of *TEP1*. *TEP1*: rs2228041 and rs1713456 are missense SNPs. *TEP1*: rs2228041 changes Arginine to Glutamine at codon 1155 and rs1713456 changes codon 1468 from Cysteine to Tyrosine. *TEP1* (telomerase‐associated protein 1, 14q11.2) gene product is a component of the telomerase enzyme complex 37. *TEP1* SNPs have been associated with the risks of bladder 38, stomach 39, prostate 40, and breast cancer 41, the prognosis of liver 42 and prostate cancer 40, and the risk of type 2 diabetes 43. Although TEP1 is a telomerase‐binding protein, previously it was shown that TEP1 is not essential for telomerase activity or telomere length maintenance in a mouse model 44. Whether TEP1 is essential for telomere length maintenance in human cells is not clear. On the other hand, TEP1 is essential for vault RNA stability and its association with the vault particle 45. Vaults are evolutionary highly conserved ribonucleoprotein particles that are associated with several cellular processes such as cell motility and differentiation 46. The associations of TEP1 SNPs with different diseases suggest that TEP1 may have general cellular functions, which when impaired, can have a broad range of physiological and pathological consequences. The molecular mechanisms underlying these associations warrant further studies. The other SNP that was associated with ovarian cancer risk was *TERT*: rs2853676. In a previous study, seven SNPs (rs2736122, rs4246742, rs4975605, rs10069690, rs2736100, rs2853676, rs7726159) in the *TERT* gene were associated with ovarian cancer risks 28. *TERT*: rs2853676 was more strongly correlated with serous ovarian cancer, consistent with our findings 28. Taken together, these data suggest that genetic variations in telomere‐maintenance genes may modulate the risks of developing ovarian cancer.

Two thirds of ovarian cancer patients die as a result of progressive disease and chemotherapy resistance. The cytotoxic effect of platinum is mediated through its interaction with DNA and formation of a variety of DNA adducts, followed by the induction of apoptosis and/or other mechanisms of cell death 47, 48. Many genes on telomere maintenance pathways have been found to be associated with chemo‐resistance to platinum *in vitro* 49, 50. In this study, we found seven SNPs significantly associated with ovarian cancer survival and four SNPs with response to platinum‐based chemotherapy. Interestingly, *TEP1*: rs10143407 and *TEP1*: rs2151753 were significantly associated with both survival and therapy response. Rs10143407 is located on the 3′UTR and rs2151753 is an intronic SNP. They may regulate the expression of TEP1 or serve as tag SNPs that are linked to causative SNPs. Further studies are needed to determine the biological mechanisms underlying the associations of these SNPs with ovarian cancer outcome. In addition to *TEP1*, the minor variant of *PINX1*: rs7826180 exhibited a nearly sevenfold increased risk of poor response for chemotherapy. *PINX1*: rs7826180 locates in an intron of *PINX1. PINX1* (*PIN2/TRF1* interacting, 8p23.1) encodes a protein of 328 amino acids and is a *TRF1*‐interacting protein. PINX1 binds to the telomerase catalytic subunit TERT and inhibits telomerase activity 51. PINX1 is a putative tumour suppressor and overexpression of PINX1 inhibits telomerase activity, shortens telomeres and induces crisis 51. A previous study showed that *PINX1* had lower expression in epithelial ovarian cancer tissues and was associated with shorter survival time 52. Our finding provided epidemiologic evidence that *PINX1* genetic variants could affect ovarian cancer outcome through telomere maintenance pathway.

There are a few limitations in our study. Firstly, the sample size was relatively small and we did not have a validation population. We used a FDR (*Q*‐value)‐based method to adjust for multiple testing and some of our findings had a *Q*‐value of less than 0.05. Nevertheless, independent validation is the ultimate means to confirm that our observations are true. Secondly, as a retrospective, hospital‐based case–control study, selection and recall bias may confound our observed associations. However, this study is a genetics‐based study and the effect of environment on genetic association is minimal as demonstrated by numerous GWAS that often had heterogeneous study designs. Thirdly, because of technical issues of iSelect SNP custom array and evolving literature, we missed some important telomere maintenance genes, such as hTERC, TIN2 and REL1, and their roles in ovarian cancer risk and outcome warrant further study. Fourthly, many of the SNPs are tagging SNPs and are not the true functional variants. The biology underlying the observed associations is unclear.

In summary, this study provides epidemiologic evidence for the associations of telomere‐maintenance gene variations with ovarian cancer risk and clinical outcome. Future studies are warranted to validate our findings and explore the biological mechanisms underlying the association of telomere maintenance gene variants with ovarian cancer risks and outcome.

## Conflicts of interest

The authors confirm that there are no conflicts of interest.

## Supporting information


**Table S1** Host and clinical characteristics of cases with ovar‐ian cancer and controls.
