# Supplementary material for: Genetic variants in telomere‐maintenance genes are associated with ovarian cancer risk and outcome
Source: J Cell Mol Med. 2016 Nov 7;21(3):510–8. doi: 10.1111/jcmm.12995 (PMC5323825; doi:10.1111/jcmm.12995)
Supplement: Supplementary file 1 — Table S1 Host and clinical characteristics of cases with ovar‐ian cancer and controls. [file JCMM-21-510-s001.docx]

**Supplementary Table 1. Host and clinical characteristics of cases with ovar­ian cancer and controls**

| **Characteristic** | **Case (%)**  **(n=417)** | **Control (%)**  **(n=417)** | ***P* value** |
| --- | --- | --- | --- |
| Age, Mean ±SD, years | 60.73±10.36 | 60.30±10.71 | 0.554 |
| Race (%) |  |  |  |
| White | 339 (81.30) | 349 (83.69) |  |
| Hispanic | 48 (11.51) | 49 (11.75) |  |
| Others | 30 (7.19) | 19 (4.56) | 0.269 |
| Clinical stage*, N (%) |  |  |  |
| Stage I | 16 (5.26) |  |  |
| Stage II | 21 (6.91) |  |  |
| Stage III | 202 (66.45) |  |  |
| Stage IV | 65 (21.38) |  |  |
| Histology*, N (%) |  |  |  |
| Serous | 197 (61.76) |  |  |
| Mixed | 72 (22.57) |  |  |
| Others | 50 (15.67) |  |  |

*Only included Caucasian patients with complete clinical information
